# Reactive electrophilic oxylipins trigger a heat stress-like response through HSFA1 transcription factors

**DOI:** 10.1093/jxb/erw376

**Published:** 2016-10-06

**Authors:** Miriam Muench, Chih-Hsuan Hsin, Elena Ferber, Susanne Berger, Martin J. Mueller

**Affiliations:** ^1^Julius-von-Sachs-Institute of Biosciences, Biocenter, Pharmaceutical Biology, University of Wuerzburg, D–97082 Wuerzburg, Germany; * Present address: Department of Pharmacology–Clinical Pharmacology Unit, University Hospital of Cologne, D-50931 Cologne, Germany

**Keywords:** 12-oxo-phytodienoic acid, acquired thermotolerance, heat stress, jasmonates, phytoprostanes, reactive electrophilic species, unfolded protein response.

## Abstract

Abiotic and biotic stresses are often characterized by an induction of reactive electrophile species (RES) such as the jasmonate 12-oxo-phytodienoic acid (OPDA) or the structurally related phytoprostanes. Previously, RES oxylipins have been shown massively to induce heat-shock-response (HSR) genes including *HSP101* chaperones. Moreover, jasmonates have been reported to play a role in basal thermotolerance. We show that representative HSR marker genes are strongly induced by RES oxylipins through the four master regulator transcription factors *HSFA1a, b, d*, and *e* essential for short-term adaptation to heat stress in Arabidopsis. When compared with Arabidopsis seedlings treated at the optimal acclimation temperature of 37 °C, the exogenous application of RES oxylipins at 20 °C induced a much weaker induction of HSP101 at both the gene and protein expression levels which, however, was not sufficient to confer short-term acquired thermotolerance. Moreover, jasmonate-deficient mutant lines displayed a wild-type-like HSR and were not compromised in acquiring thermotolerance. Hence, the OPDA- and RES oxylipin-induced HSR is not sufficient to protect seedlings from severe heat stress but may help plants to cope better with stresses associated with protein unfolding by inducing a battery of chaperones in the absence of heat.

## Highlight

Electrophilic oxylipins trigger a heat-shock-like response in the absence of heat through the canonical heat-shock transcription factor A1, thereby helping to cope with stresses associated with protein damage.

## Introduction

Plants are able to acclimate to transient severe heat stress by triggering an evolutionary conserved, genetically programmed heat-shock response (HSR). The HSR is characterized by expression of a battery of heat shock proteins (HSPs), many of which are molecular chaperones involved in correct native folding and/or assembly of other proteins (Finka *et al.*, 2011). In Arabidopsis, the HSR is induced by temperatures above 32 °C and reaches a maximum at 37 °C, a temperature which can be tolerated for several days (Yeh *et al.*, 2012). Even after a short exposure to the optimal acclimation temperature (1–2 h at 37 °C), Arabidopsis seedlings are able to survive an otherwise lethal heat shock at 45 °C for 2 h. This phenomenon has been termed short-term acquired thermotolerance (SAT) and involves the rapid activation of the four constitutively expressed heat shock factors A1 (*HSFA1 a, b, d, e*) (Liu *et al.*, 2011; Yoshida *et al.*, 2011) that subsequently activate a series of inducible HSF ultimately leading to the rapid accumulation of heat shock proteins (HSPs) (Saidi *et al.*, 2011). The four master *HSFA1* genes regulate the expression of 65% of all heat-up-regulated genes and are essential for heat acclimation (Liu *et al.*, 2011). Among the HSPs, HSP101 has been shown to play an essential role in basal and acquired thermotolerance in *A. thaliana* (Queitsch *et al.*, 2000; Larkindale *et al.*, 2005). The loss of other HSPs also results in at least reduced thermotolerance (Yamada *et al.*, 2007; Dafny-Yelin *et al.*, 2008; Su and Li, 2008).

The primary heat sensors are still unclear and activation of HSFA1-dependent and independent responses towards heat may involve multiple signalling pathways (Mittler *et al.*, 2012). In the classical model for heat-shock response activation, heat-induced unfolded cytosolic proteins bind to molecular chaperones and trigger a release of inhibitory HSP90 and HSP70 proteins from the complex with the constitutive HSFA1s. Subsequently, free HSFA1s trimerize, undergo phosphorylation, bind to HSR promotors, and activate the HSR (Scharf *et al.*, 2012).

Notably, a heat-shock-like response leading to enhanced thermotolerance can also be induced by different small molecules in the absence of heat. For instance, HSP90 inhibitors such as geldanamycin and radicicol have been shown to induce a heat-shock-like response and to enhance thermotolerance in Arabidopsis and *Physcomitrella* (Yamada *et al.*, 2007; Saidi *et al.*, 2009). In addition to established HSP90 inhibitors, reactive oxygen species (ROS), reactive nitrogen species (RNS), and reactive electrophile species (RES) have been shown to covalently modify protein thiols of an array of proteins including HSP90s and HSP70s in several organisms and to induce a heat-shock-like response (Mollapour and Neckers, 2012; Wang *et al.*, 2012; West *et al.*, 2012).

In plants, several RES including 2-hexenal, 12-oxo-phytodienoic acid (OPDA) and A_1_-phytoprostanes (PPA_1_) can be formed endogenously by enzymatic or non-enzymatic lipid peroxidation and levels of these RES increase after a variety of stresses (Farmer and Mueller, 2013). Exogenous exposure of Arabidopsis leaves to (*E*)-2-hexenal strongly and rapidly induced HSPs and was shown to increase thermotolerance (Yamauchi *et al.*, 2015).

In addition, cyclopentenones such as OPDA, a jasmonic acid precursor, and PPA_1_ have also been shown to increase the expression of inducible HSFs and HSPs dramatically (Taki *et al.*, 2005; Mueller *et al.*, 2008). OPDA, as well as its metabolite jasmonic acid (JA), accumulate when Arabidopsis leaves are exposed to the acclimation temperature at 37 °C (Clarke *et al.*, 2009). However, only OPDA and not the non-RES lipid JA trigger the accumulation of HSPs (Mueller *et al.*, 2008). To a large part, RES cyclopentenone-induced genes are dependent on TGA transcription factors. However, the induction of HSFs and HSPs by cyclopentenones do not involve TGA factors (Mueller *et al.*, 2008) and may be dependent or independent on the activation of HSFA1s and/or the putative OPDA receptor protein cyclophilin 20-3 (CYP20-3). Upon binding of OPDA, CYP20-3 has been shown to bind and activate serine acetyltransferase 1 (SAT 1) that is involved in a hetero-oligomeric cysteine synthase complex. OPDA signalling through this complex leads to the accumulation of thiol metabolites, a build-up of cellular reduction potential, and the expression of a subset of OPDA-responsive genes including a small HSP (Park *et al.*, 2013).

In this study, we investigate the role of putative signalling components in the cyclopentenone-induced heat-shock-like response and clarify the functional significance of OPDA in different thermotolerance assays.

## Materials and methods

### Plant growth conditions and chemicals

*Arabidopsis thaliana* wild-type ecotypes ‘Columbia-0’ (Col-0) and ‘Wassilewskija’ (WS) as well as the mutant lines *hsfA1 abde* [provided by K Yamaguchi-Shinozaki (Yoshida *et al.*, 2011)], *hsfA2* (SALK_008978), *cyp20-3*, and *sat1* [provided by CB Lawrence (Park *et al.*, 2013)], and the *dde2-2* [provided by B Keller (von Malek *et al.*, 2002)] were grown in a growth chamber under an 8/16 h short-day cycle at 22 °C (100 µE)/20 °C. Plants were either grown in liquid or on solid medium comprising sterile MES buffered Murashige and Skoog (MS) medium, pH 5.7 (Duchefa Biochemie BV, Haarlem, Netherlands) and 3% sucrose without (liquid medium) or with 0,9% agar (agar plates), respectively. Wounding experiments were performed with plants grown on soil by wounding each side of a leaf three times with forceps without damaging the midvein. Geldanamycin, celastrol, radicicol, and PGA_1_ were purchased from Cayman Chemical (Ann Arbor, USA). OPDA was synthesized from linolenic acid using linseed acetone powder and purified by HPLC as described in Park *et al.* (2013).

### Gene expression analysis

Extraction of total RNA from plant material (eight 10-d-old-seedlings/sample; three replicates) was performed by using peqGOLD TriFast^TM^ reagent (PEQLAB). The RNA concentration was determined spectrophotometrically. The remaining DNA was removed using RNase-free DNase I (Fermentas, Waltham, USA). RNA was reverse transcribed to cDNA using RNA M-MLV reverse transcriptase (Promega, Madison, USA). Real-time PCR was performed using the ABsolute SYBR Capillary Mix (Thermo Fisher Scientific, Waltham, USA) and a CFX 96 Real-Time System C1000 Thermal Cycler (Bio-Rad, Hercules, USA). The primers used (TIB MOLBIOL, Berlin, Germany) were as follows: *HSP101* (At1G74310): forward: 5′-TGAGCTAGCTGTGAATGCAG-3′, reverse: 5′-TCAACTGGTCAACAGCCAAA-3′; *HSFA2* (AT2G26150): forward: 5′-CAGCAAGGATCTGGGATGTC-3′, reverse: 5′-GCTGTT GCCTCAACCTAACT-3′; *DREB2A* (At5g05410): forward: 5′-AGGGTCGAAGAAGGGTTGTA-3′, reverse: 5′-CAGCTT CTTGAGCAGTAGGG-3′; *HSP26.5* (At1g52560): forward: 5′-TGT GAAAGAGGTTTGGTCGG-3′, reverse: 5′-TGTTACGCCAGA GGCTTTTT-3′. The annealing temperature for all primers was 59 °C. Gene expression relative to *AtSAND* (At2g28390) (forward: 5′-AACTCTATGCAGCATT-3′, reverse: 5′-GGTGGTACTAGCACAA-3′) was measured by using the delta cycle threshold method (Pfaffl, 2001).

### Protein isolation and dot blot analysis

For protein isolation, samples (eight seedlings per sample) were shock frozen in liquid nitrogen and ground in a ball mill at 20 Hz for 2 min (Retsch, Haan, Germany). Proteins were extracted with 50 µl HEPES buffer (containing 10 mM HEPES-KOH (pH 7.6), 5 mM sucrose, and 5 mM MgCl_2_) in a vortex mixer for 2 min. Cell debris was removed by centrifugation at 4 000 *g* (4 °C). Protein levels in the samples were quantified with the Bradford assay (Bradford, 1976). Extracted proteins (20–50 µg) were blotted on to a nitrocellulose membrane using a dot blot apparatus (Bio-Dot^TM^ Apparatus; Bio-Rad laboratories, Munich, Germany). The blot was blocked with 3% non-fat dried milk in TRIS-buffered saline with Tween20 (TBST) for 2 h at room temperature. Thereafter, the membrane was incubated with a polyclonal rabbit anti-HSP101 antibody (Product No: AS07 253; Lot 1006; Agrisera, Vännäs, Sweden) at a dilution of 1:1 000 in TBST including 3% non-fat dried milk powder. The immune complexes were detected using horseradish peroxidase conjugated to a secondary antibody (1:20 000) and chemoluminescence detection was accomplished using the Clarity^TM^ Western ECl substrate and the ChemiDoc^TM^ MP Imaging System using the software Image Lab^TM^ Software, Version 5.0 (Bio-Rad, laboratories, Munich, Germany). For normalization, a dilution series of protein extracts obtained from seedlings treated at 37 °C for 4 h (set to 100%) were compared with the HSP101 levels in the other samples. Relative HSP101 levels were expressed as a percentage of the 37 °C treated samples.

### Thermotolerance assays

For testing seedling survival, thermotolerance tests were performed as described by Hong and Vierling (2000) with small modifications. Seedlings were grown on agar plates for 6 d and then transferred into sterile, liquid MS medium. Experiments were performed on day 7 at growth stage 1.0 (Boyes *et al.*, 2001). Seedlings were left at 22 °C (controls) or received a heat shock at 45 °C for 80 min with or without prior heat acclimation (37 °C for 2 h followed by 2 h at 22 °C). To test the effect of chemicals (HSP90 inhibitors or oxylipins dissolved in DMSO), the acclimation phase at 37 °C was replaced by a chemical treatment or a solvent control (the final DMSO concentration did not exceed 2.8%, v/v) for 2 h at 22 °C prior to the heat shock at 45 °C. After the treatments, seedlings were grown at 22 °C for 5–6 d and the survival of the seedlings was then evaluated as judged by complete growth inhibition and cotyledon bleaching.

For determination of the survival rate of Col-0 versus *dde2* plants, seedlings were grown on agar plates for 4–7 d under standard short-day conditions (growth stage 1.0). Seedlings were treated with a heat shock at 45 °C for 2 h without prior acclimation (HS) or pretreated at 37 °C for 2 h followed by 2 h at 22 °C to acquire thermotolerance before receiving the heat shock at 45 °C for 2 h (AT).

Root growth assays were performed as described by Larkindale *et al.* (2005) with small modifications. Briefly, seedlings were grown on MS agar plates for 4–7 d under standard short-day conditions (growth stage 1.0). Heat treatments were performed as described above in the light. After returning to normal growth conditions, root elongation was evaluated after 5 d.

Hypocotyl elongation assays were performed as described previously (Hong and Vierling, 2000; Queitsch *et al.*, 2000). Seedlings (4–7 d, growth stage 1.0) grown on MS agar plates received the heat treatments described above. After an additional 5 d in the dark, hypocotyl elongation was determined.

### Jasmonate analysis

For the analysis of jasmonates, shock-frozen 14-d-old seedlings were ground by mortar and pestle. The frozen plant powder (50–200 mg) was extracted and analysed by ultra-high-performance liquid chromatography–tandem mass spectrometry as described by Grebner *et al.* (2013)

### *In silico* gene expression analysis

The microarray data used in this study for *in silico* analysis can be found in the Gene Expression Omnibus at the National Center for Biotechnology Information (NCBI: accession numbers GSE26266 and GSE10749) (Dueckershoff *et al.*, 2008; Liu *et al.*, 2011).

## Results

### The cyclopentenone lipids OPDA and prostaglandin A_1_ strongly induce HSR marker genes through HSFA1

**Fig. 1.**
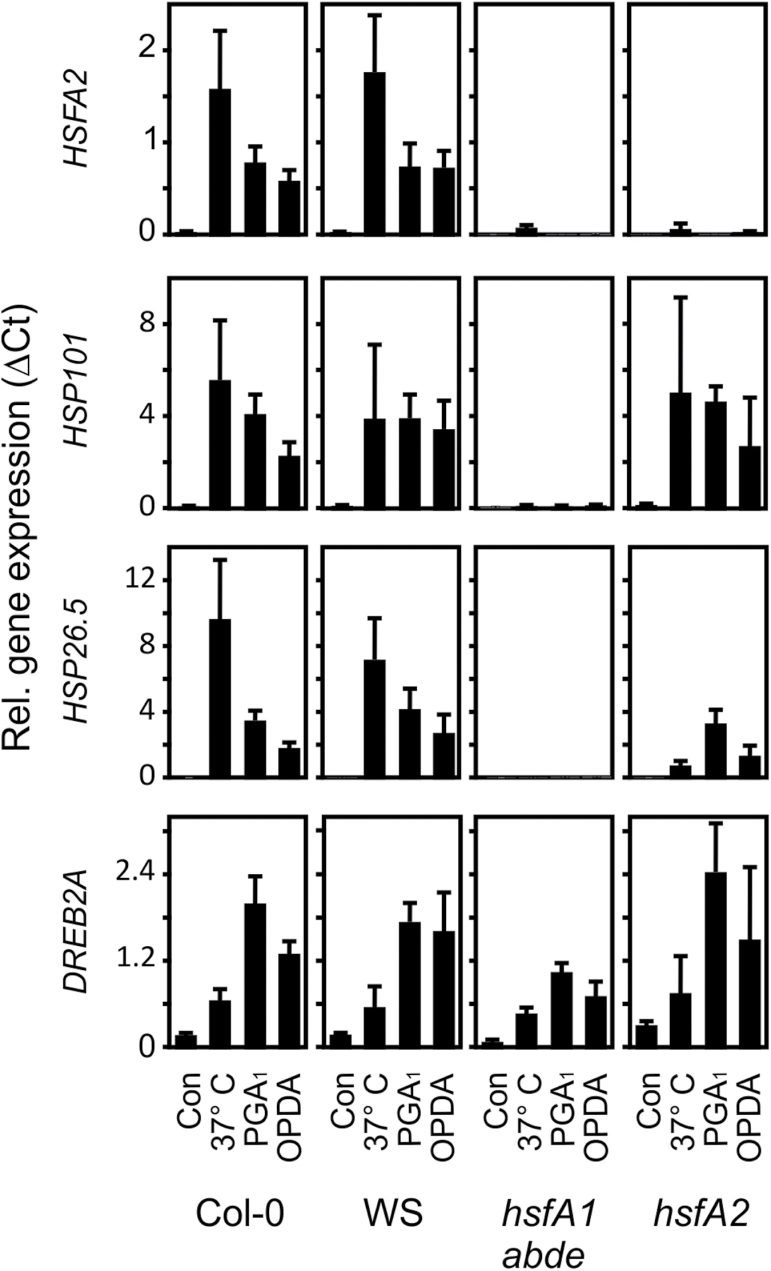
Induction of heat-responsive genes by moderate heat and RES cyclopentenones in wild-type (Col-0, WS), *hsfA1 abde*, and *hsfA2* plants. Seedlings were treated with cyclopentenones (PGA_1_ or OPDA, 75 µM) or moderate heat (37 °C) for 4 h and gene expression was analysed by qPCR. Expression was normalized to *AtSAND.* Data represent means ±SD, *n*=3.

Moderate heat triggers an HSR that is dependent on the four constitutive HSFA1 transcription factors (a, b, d, and e) and characterized by a dramatic induction of heat shock marker genes including the inducible transcription factors *HSFA2* and *dehydration-responsive element-binding protein 2A* (*DREB2A*) as well as *HSP101* and *HSP26.5*. In order to compare the activation of the marker genes by moderate heat and cyclopentenones, we treated seedlings with moderate heat (37 °C), OPDA, and prostaglandin A_1_ (PGA_1_, a commercially available analogue of A_1_-phytoprostanes) for 4 h. In addition, we tested the expression of the HSR marker genes in the *hsfA1 abde* quadruple and *hsfA2* mutant plants. Since the *hsfA1* quadruple mutant was generated by crossing single mutants from the ecotypes Col-0 and WS, we used both wild types as controls (Fig. 1).

In the wild types, Col-0 and WS, cyclopentenone-induced expression of *HSFA2*, *HSP101*, and *HSP26.5* was about 30–90% of the expression of these genes after 37 °C for 4 h. Notably, HSP101 that has been shown to be essential and sufficient to establish acquired thermotolerance was strongly induced at 37 °C (68-fold) as well as by PGA_1_ (50-fold) and OPDA (28-fold) in *A. thaliana* Col-0 seedlings. The expression of the *DREB2A* gene is induced by heat shock and dehydration via *HSFA1*-dependent and -independent signalling pathways, respectively (Yoshida *et al.*, 2011). In contrast to the other marker genes, induction of *DREB2A* by cyclopentenones was even stronger than by the 37 °C treatment in the wild types.

Cyclopentenone-induced expression of *HSFA2*, *HSP101*, and *HSP26.5* but not of *DREB2A* was strictly dependent on the four HSFA1 master regulator genes of the HSR. In addition, the inducible transcriptions factor HSFA2 was not found to be required for up-regulation of *HSP101* and *DREB2A* by heat and RES oxylipins. However, HSFA2 was required for the full up-regulation of the small *HSP26.5* gene by heat but not by RES oxylipins.

Hence, the results indicate that HSFA1s but not HSFA2 are essential for the induction of heat-responsive HSPs by OPDA and PGA_1_.

### *The putative OPDA receptor* CYP20-3 *and its interacting partner* SAT1 *are not essential for induction of the heat-shock-like response by cyclopentenones*

**Fig. 2.**
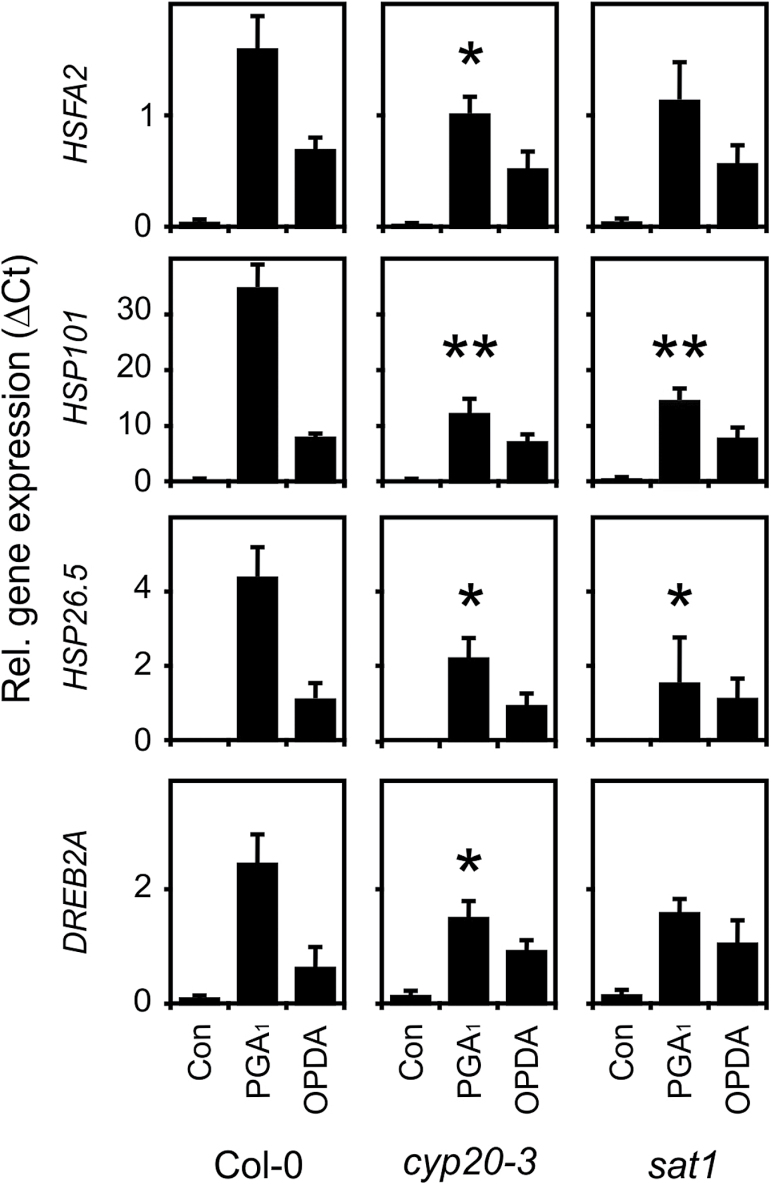
Induction of heat-responsive genes by RES cyclopentenones in wild-type (Col-0), *cyp20-3*, and *sat1* plants. Seedlings were treated with cyclopentenones (PGA_1_ or OPDA, 75 µM) for 4 h and gene expression was analysed by qPCR. Expression was normalized to *AtSAND.* Data represent means ±SD, *n*=3. Significant differences between the mean values of the mutants versus the wild type are indicated by asterisks using Students’s *t* test (*, *P* <0.05; **, *P* <0.01).

Recently, it has been shown that CYP20-3 relays an OPDA signal in plastid Cys biosynthesis thereby enhancing the plastid redox capacity and activating a subset of OPDA responsive genes (Park *et al.*, 2013). Upon OPDA binding to CYP20-3, a hetero-oligomeric Cys synthase complex involving SAT1 and *O*-acetylserine (thiol)lyase B is formed in chloroplasts. To determine the significance of CYP20-3 and SAT1 for the induction of heat-responsive genes by cyclopentenones, we analysed marker gene expression of the heat-shock response in mutants deficient in these genes. As shown in Fig. 2, OPDA-induced expression of *HSFA2, HSP101, HSP26.5*, and *DREB2A* was not significantly altered in *cyp20-3* and *sat1* plants while the response to PGA_1_ was reduced but not abolished. Although OPDA and PGA_1_ are expected to display similar electrophilic properties and thiol reactivities as well as membrane permeability, their reactivity with different thiols in proteins is also dependent on the structure-dependent affinity to the protein binding site. Therefore, even structurally related RES may display differences in their biological activity (reviewed in Mueller and Berger, 2009). Hence, CYP20-3 and SAT1 appear to contribute but are not essential for cyclopentenone-induced expression of several HS-genes.

### Kinetics of HSP101 gene expression and protein accumulation in response to chemical inducers and moderate heat

**Fig. 3.**
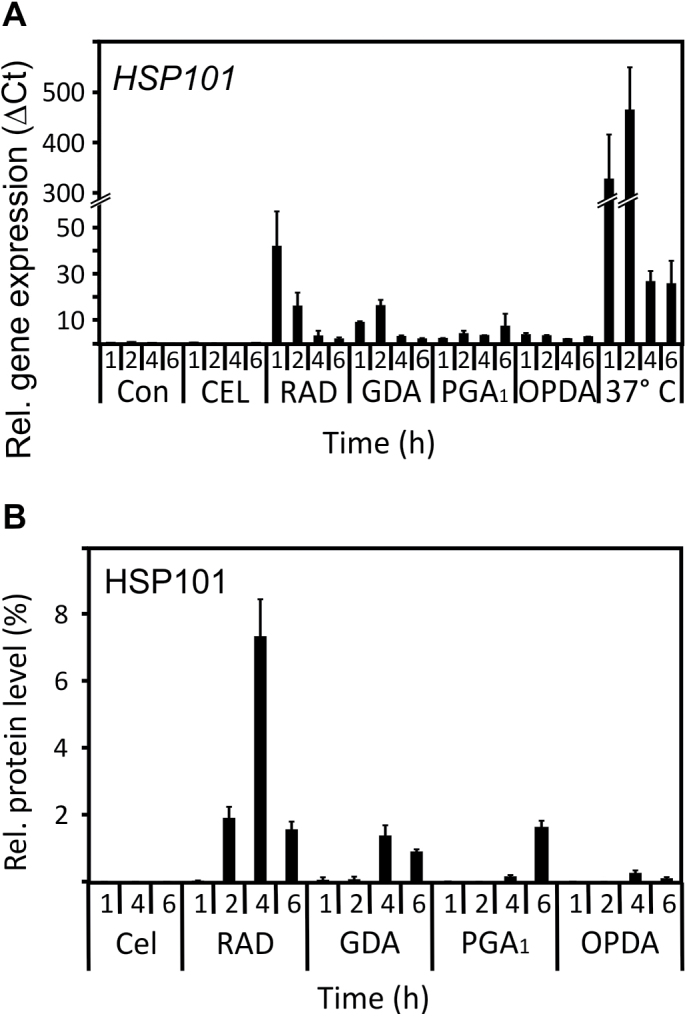
Time-course of *HSP101* gene expression and HSP101 protein accumulation after treatment with chemical inducers or moderate heat. (A) Seedlings were treated with the HSP90-inhibitors celastrol (CEL, 20 µM), radicicol (RAD, 50 µM), geldanamycin (GDA, 50 µM), and the RES cyclopentenones (PGA_1_ and OPDA, 75 µM) for the times indicated. *HSP101* mRNA levels were determined by qPCR and normalized to *AtSAND*; data represent means ±SD, *n*=3. (B) Relative HSP101 protein levels were evaluated by dot blot analysis and shown as a percentage of seedlings subjected to 37 °C heat treatment for 4 h. Data represent means of three technical replicates of a representative experiment ±SD.

The best characterized chemical inducers of an HS-like response are HSP90 inhibitors such as radicicol (RAD), geldanamycin (GDA), and celastrol (CEL) that have been shown to induce acquired thermotolerance in Arabidopsis or yeast (Yamada *et al.*, 2007; Trott *et al.*, 2008). In Arabidopsis, the expression of HSR-marker genes in response to RAD and GDA at 22 °C was comparable with exposure to moderate heat (37 °C) when tested after 6 h (Yamada *et al.*, 2007). However, moderate heat typically induces a rapid and transient increase of expression of HS-marker genes with a maximum about 1–2 h after heat exposure followed by a dramatic and rapid decline in HSR gene expression. In order to compare the effect of HSP90 inhibitors and RES cyclopentenone lipids with moderate heat, we determined the time-course of *HSP101* expression. By far the strongest effect on gene expression was observed after 2 h at 37 °C while the chemical inducers were well below 4% (GDA, PGA_1_, and OPDA) or 9% (RAD) of the highest heat-induced expression level at any time point (Fig. 3A). Celastrol did not induce a significant increase in *HSP101* transcription.

To confirm these results at the protein level, relative HSP101 protein accumulation after chemical treatments was determined by dot blot analysis (Fig. 3B). For comparison, the HSP101 protein level after 4 h of moderate heat stress at 37 °C was defined as 100%. Among the chemicals, radicicol induced the highest relative HSP101 accumulation (7.3% after 4 h) while the other chemicals induced not more than 2% of the HSP101 levels observed after moderate heat stress. Again, CEL did not lead to an accumulation of HSP101.

Hence, we observed a strong induction of *HSP101* gene expression and HSP101 protein accumulation by radicicol, geldanamycin, PGA_1_, and OPDA relative to the untreated control seedlings (Figs 1, 2), however, induction of *HSP101* was very low compared with the 37 °C heat stress (Fig. 3).

In order to clarify, if the induction of HS-responsive genes by HSP90 inhibitors and RES cyclopentenone lipids in the absence of heat is sufficient to confer acquired thermotolerance, we performed a standard acquired thermotolerance assay. Arabidopsis seedlings (7-d-old) were either acclimated at 37 °C for 2 h and allowed to recover at 22 °C for 2 h or treated with the chemicals for 4 h at 22 °C. Thereafter, seedlings received a heat shock (45 °C) for 80 min and were returned to the growth chamber at 22 °C. The survival of seedlings was examined 6 d after the heat shock at 45 °C. All seedlings that were treated with the chemicals (at the concentrations indicated in Fig. 3) or a mock treatment (solvent control) at 22 °C did not survive the heat shock at 45 °C while all hte seedlings that were acclimated at 37 °C prior to the heat shock survived (data not shown). Hence, the induction of HSR-genes by HSP90-inhibitors and RES cyclopentenones was not sufficient to confer a chemically-induced thermotolerance, at least under the standard acquired thermotolerance assay conditions.

### Moderate heat- and wound-induced endogenous jasmonates do not trigger HSP101 expression

**Fig. 4.**
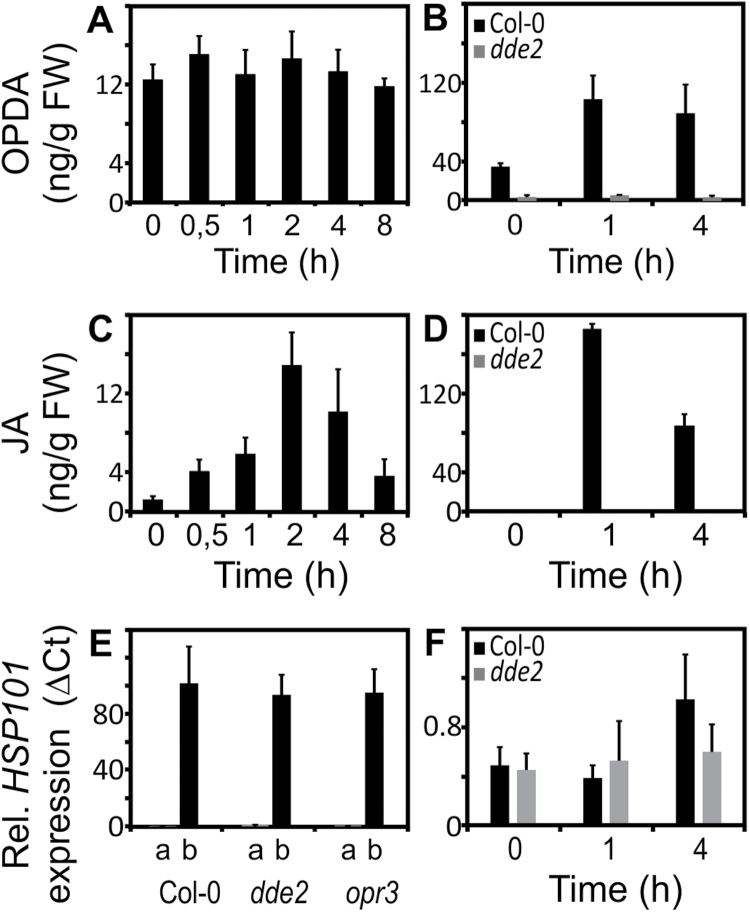
Heat- and wound-induced jasmonate accumulation and *HSP101* expression. Time-course of jasmonate accumulation in seedlings shifted to 37 °C (A, C) and wounded wild-type and *dde2* leaves from 6-week-old plants (B, D). Relative *HSP101* expression (as determined by qPCR and normalized to *AtSAND* expression) in wild-type and mutant seedlings after 1 h at the temperatures indicated (E) or after wounding (F). Data represent means ±SD, *n*=3.

Since exogenously administered OPDA strongly induced HSPs, endogenously formed OPDA or an OPDA metabolite may potentially be involved in the heat-shock response. To clarify if endogenous jasmonates accumulate in response to moderate heat, we determined the levels of OPDA and JA after moving the seedlings from the normal growth temperature (22 °C) to the optimal acclimation temperature at 37 °C. However, OPDA levels remained constant for at least 8 h at 37° C and the JA levels showed only a weak and transient accumulation with a maximum after 2 h (Fig. 4A, C). Moreover, heat-induced expression of the *HSP101* gene was not compromised in the *delayed dehiscence 2* (*dde2*) or the *12-oxophytodienoic acid reductase 3* (*opr3*) mutant lines defective in the biosynthesis of all jasmonates or all jasmonates downstream of OPDA including JA, respectively (Fig. 4E).

Since an exogenous application of OPDA clearly triggered an HSR-like response and HSPs are regarded as beneficial for general stress resistance (Farmer and Mueller, 2013), we also addressed whether endogenously generated OPDA induces *HSP101* expression. Wounding is a trigger for endogenous OPDA and JA biosynthesis and we observed that both jasmonates accumulated after wounding of wild-type but not *dde2* leaves (from 6-week-old Arabidopsis plants) with tweezers (Fig. 4B, D). The wound-induced endogenous accumulation of jasmonates was much higher than after heat treatment. However, wound-induced jasmonates did not strongly induce the expression of the *HSP101* gene in both wild-type and *dde2* plants (Fig. 4F) suggesting that wound-induced OPDA does not trigger a strong HSR-like response.

### Jasmonates are not involved in establishment of acquired thermotolerance

**Fig. 5.**
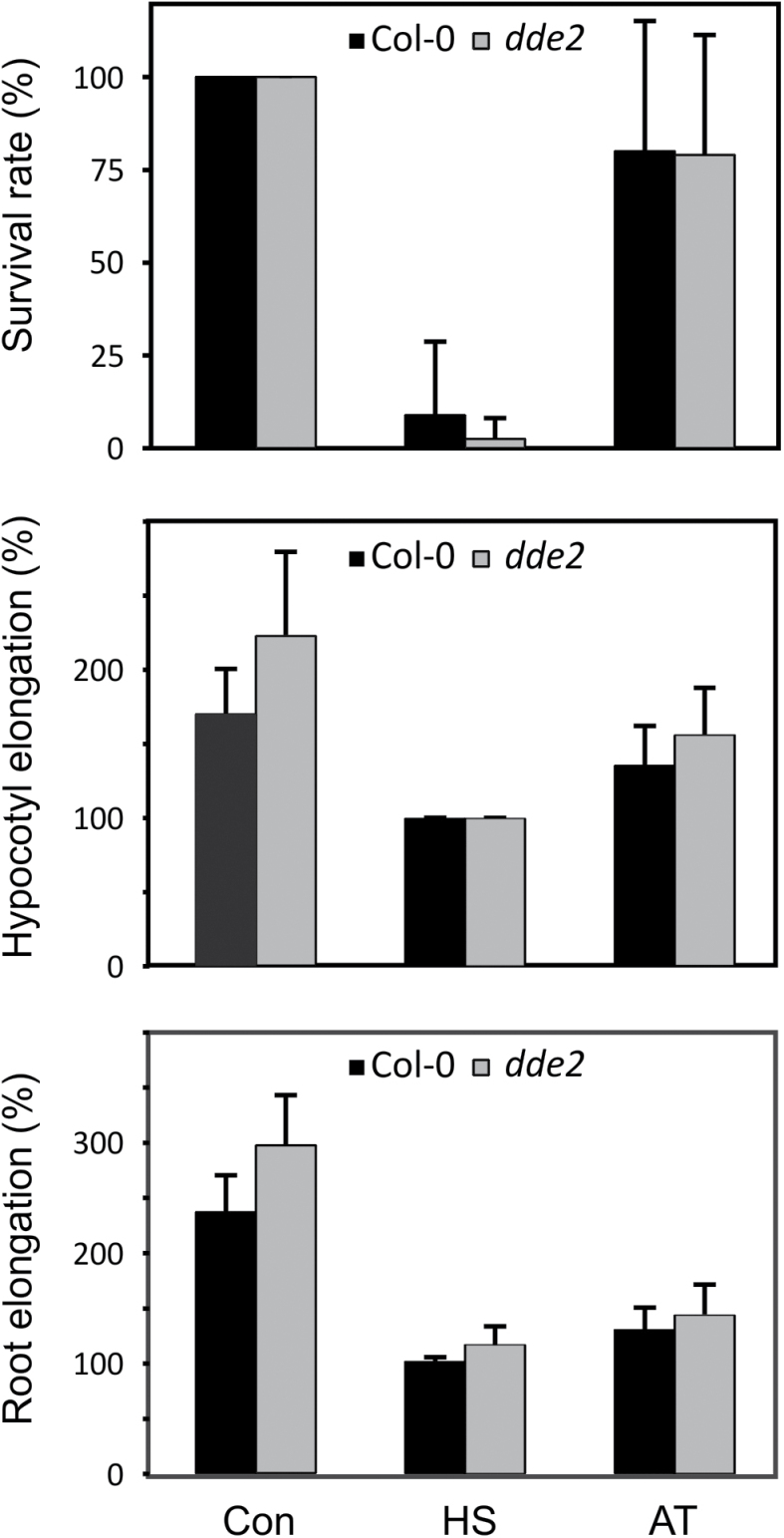
Standard acquired thermotolerance assays. Wild-type (black bars) and *dde2* mutant (grey bars) seedlings were grown on agar plates at 22 °C. Seedlings were left at 22 °C (Con), treated with a heat shock at 45 °C for 2 h without prior acclimation (HS) or pretreated at 37 °C for 2 h followed by 2 h at 22 °C to acquire thermotolerance before receiving the heat shock at 45 °C for 2 h (AT). After the heat treatments, seedlings were returned to 22 °C. Survival, as judged by the ability of the seedlings to develop green leaves, was determined 5 d after the heat treatment. The hypocotyl and root length determined before the treatments was set to 100% and compared with the length of these organs 5 d after the treatments.

In addition, we tested if the jasmonate-deficient *dde2* mutant was compromised in establishing acquired thermotolerance after heat acclimation at 37 °C. Three standardized short-term acquired thermotolerance assays were performed: the seedling survival, the hypocotyl elongation, and the root elongation thermotolerance assay. In all thermotolerance assays, mutant plants displayed normal acquired thermotolerance compared with the wild type indicating that jasmonates are not involved in the establishment of short-term acquired thermotolerance (Fig. 5).

## Discussion

A wide array of structurally highly diverse RES compounds, including cyclopentenone oxylipins such as OPDA and A_1_-phytoprostanes or alkenales such as 3-hexenal, have been shown to induce a battery of heat-shock genes.

When comparing the heat-shock-gene-inducing effect of RES compounds with heat 3–4 h after the treatments, gene induction often appears to be dramatic and in the same order of magnitude. This is also the case for cyclopentenone oxylipins (Fig. 1). After a heat shock, however, heat-responsive genes typically reached their highest expression level 1–2 h after the onset of heat followed by a rapid and dramatic decline (1–2 orders of magnitude) in gene expression (Fig. 3). By contrast, the plant response towards exogenously administered RES compounds appears to be slower in onset and the expression of most RES-responsive genes, including HS genes, remain elevated for several hours after the treatment. Comparison of the highest expression levels of representative HS genes (Fig. 3A) revealed that the RES-oxylipin-induced HS gene expression levels were well below 2% of the highest levels during moderate heat stress and this also translated into dramatically lower protein levels, at least in the case of HSP101 (Fig. 3B).

The induction of more than 65% of all the 1042 genes up-regulated by heat more than 2-fold (Finka *et al.*, 2011), including most *HSF*s and *HSP*s, is mediated by the four master regulators of the heat-shock response, the *HSFA1 a*, *b*, *d*, and *e* (Liu *et al.*, 2011). We show that induction of *HSFA2*, *HSP26.5*, and *HSP101* by cyclopentenone oxylipins is also strictly dependent on these master regulators (Fig. 1) while the heat- and stress-responsive factor DREB2A is not dependent on HSFA1 and appears to be regulated through other signalling pathways. When comparing the published transcriptome analyses of heat and OPDA-induced genes, a large number of heat-responsive and HSFA1-dependent genes were not found to be induced by exogenously administered OPDA. Out of the 348 genes up-regulated more than 3-fold by moderate heat at 37 °C for 1 h (Liu *et al.*, 2011), only a subset of 30 genes is also up-regulated by 75 µM OPDA (out of a total of 444 OPDA up-regulated genes) after 4 h (Mueller *et al.*, 2008) more than 3-fold. From the 30 genes up-regulated by heat and OPDA, 27 genes have previously been identified to be dependent on the HSFA1 master regulators after moderate heat and belong to the most strongly up-regulated heat-responsive genes (see Supplementary Table S1 at *JXB* online). We hypothesize that the apparent lack of induction of many HSFA1-dependent genes (>3-fold) after OPDA treatment is due to the comparably weak heat-shock-like response induced by OPDA.

The mechanism of how RES oxylipins or heat activates a heat-shock response via HSFA1 is not known. According to the widely accepted ‘chaperone titration model’ for heat-shock activation, the presence of unfolded proteins causes the release of the HSP70 and HSP90 chaperones from their constitutive inhibitory complex with HSFA1 monomers to bind unfolded proteins, while the free HSFs subunits trimerize, become phosphorylated, bind to HSR promotors, and activate the heat-shock response (Neef *et al.*, 2011). Interestingly, this scenario implies that the cell does not recognize temperature per se. Rather, it suggests that the heat-shock response is triggered by unfolded proteins that are the result of a variety of stresses, including RES, oxidative stress (ROS), heavy metals, ethanol or other toxic substances (Richter *et al.*, 2010). In Arabidopsis, it has been shown that HSP70 and HSP90 indeed interact with HSFA1 and repress the activation of the heat-shock response in the absence of heat (Yamada *et al.*, 2007; Ohama *et al.*, 2016).

However, it has been questioned if the level of unfolded proteins regulates the dissociation of the inhibitory complex and alternative heat sensing mechanisms have been suggested (Mittler *et al.*, 2012; Ohama *et al.*, 2016). With respect to the activation of the heat-shock response by RES, it has been proposed that RES bind to and covalently modify HSP70 and/or HSP90 causing a destabilization of the inhibitory complex and the release of HSF (Scroggins and Neckers, 2007; Jacobs and Marnett, 2010). In human HSP90, modification of Cys 572 by the RES lipid peroxidation product 4-hydroxynonenal has been observed in a rat model of chronic alcohol liver disease and shown to impair its chaperone activity *in vitro* (Carbone *et al.*, 2005). Moreover, inhibition of the HSP90 chaperone activity by HSP90 inhibitors such as GDA or RAD has been shown not only to induce heat-shock genes but also to confer short-term acquired heat tolerance in a variety of organisms including *Arabidopsis thaliana* (Yamada *et al.*, 2007). Notably, the HSP70 SSA1 has been identified in yeast as a sensor for the heat-shock response by thiol-reactive compounds (Wang *et al.*, 2012). Involvement of HSP90 in the activation of the OPDA-dependent induction of heat-shock genes has been suggested, however, the primary OPDA-sensing protein still remains elusive (Masuda *et al.*, 2014). Ultimately, however, OPDA and other RES compounds appear to trigger a release of HSFA1 from the inhibitory complex with HSP70 and HSP90 leading to the activation of a heat-shock-like response.

To this end we addressed the question whether cyclopentenones such as OPDA can induce a heat-shock-like response that is sufficient to confer heat tolerance. Recently, it has been shown that exogenously applied (*E*)-2-hexenal and other short-chain alkenals induce a heat-shock-like response that was sufficiently strong to confer acquired thermotolerance enabling Arabidopsis seedlings to survive a 45 °C heat shock for 2 h which is lethal to control seedlings (Yamauchi *et al.*, 2015). However, when we tested the effect of seedling pretreatment with RES cyclopentenone lipids and HSP90 inhibitors under the conditions used to demonstrate the thermotolerance-inducing effect of HSP90 inhibitors (Yamada *et al.*, 2007), we did not observe an increase in thermotolerance after the chemical treatments in the three standard thermotolerance assays while standard heat acclimation (37 °C for 2 h) always and robustly protected the seedlings from lethal heat stress (45 °C for 80–120 min). While all these chemicals induced heat-shock genes, the effect appeared not to be sufficient to confer thermotolerance at least under our test conditions. Although we tried several variations of the thermotolerance assays, we never succeeded in inducing acquired thermotolerance with cyclopentenones over a wide concentration range. On the other hand, the lipid RES 3-hexenal has been shown to elicit a heat-shock-like response that is sufficient to confer thermotolerance (Yamauchi *et al.*, 2015).

Most or even all RES induce heat-shock genes as well as a common suite of detoxification and stress genes, however, the response varies with respect to the magnitude of gene regulation. Moreover, microarray analyses also revealed that the response towards different RES displays differences with respect to the regulated genes, indicating that structurally different RES differentially activate signalling pathways. Although it is generally accepted that biological activity is dependent on the thiol reactivity of a particular RES species, the RES molecular structure may strongly modulate the biological response. For instance, the RES molecules lipophilicity has an impact on its membrane permeability and metabolic stability. Besides RES reactivity and lipophilicity, the molecules structure and conformation may also affect the RES affinity to target proteins thereby facilitating or preventing covalent binding to thiols on proteins (reviewed in Mueller and Berger, 2009).

Previous reports have suggested that jasmonates are important for basal thermotolerance (Clarke *et al.*, 2009). The *coi1-1* mutant deficient in jasmonate perception is more susceptible to mild chronic heat stress (38 °C), however, it has not been reported whether endogenous jasmonates play a role in acquired thermotolerance induced by mild heat (37 °C). In Arabidopsis seedlings exposed to 37 °C, we did not observe an increase in endogenous OPDA levels during the 8 h of heat stress and jasmonic acid levels displayed a small transient increase 2 h after the onset of heat (Fig. 4). Moreover, the *dde2* mutant, unable to synthesize OPDA and JA, displayed normal heat-induced *HSP101* expression (Fig. 4) and behaved as the wild type in the thermotolerance assays (Fig. 5).

We conclude that exogenously administered RES cyclopentenone lipids trigger a HSFA1-dependent heat-shock-like response that is—in contrast to other lipid RES such as 3-hexenal— not strong enough to confer acquired thermotolerance in Arabidopsis seedlings. We could not find any evidence supporting the idea that endogenous jasmonates play a role in short-term acquired thermotolerance. However, severe heat stress is possibly one of the strongest protein denaturating stress conditions and therefore requires an extremely rapid and strong induction of the HSR. We propose that the cyclopentenone-induced HSR may be relevant under less severe stresses. The accumulation of endogenous OPDA and other RES lipids such as phytoprostanes and alkenals has been observed after several abiotic and biotic stresses associated with the partial unfolding of proteins and the induction of heat-shock genes (Mueller *et al.*, 2008; Yamauchi *et al.*, 2015). In addition, RES such as methyl glyoxal and isothiocyanates derived from carbohydrate and secondary metabolism may also contribute to the stress-induced cellular RES load. To this end, we previously showed that cyclopentenone RES not only accumulated after heavy metal stress but also increased resistance against heavy metal intoxication (Loeffler *et al.*, 2005). Thus, RES cyclopentenone lipids clearly contribute to the total stress-induced RES pool and act in concert with other inducible RES. Hence, OPDA and structurally related cyclopentenones may help to cope with unfolded protein stress by inducing chaperones involved in refolding damaged proteins.

## Supplementary data

Supplementary data can be found at *JXB* online.

Table S1. In silico analysis of OPDA- and heat-up-regulated genes.

Supplementary Data

## Abbreviations:

CEL: celastrol
CYP20-3: cyclophilin 20-3
dde2: *delayed dehiscence 2*
DREB2A: dehydration-responsive element-binding protein 2A
GDA: geldanamycin
JA: jasmonic acid
JA-Ile: jasmonic acid isoleucine
HSFA1/A2: heat shock transcriptions factors A1/A2
HSP: heat-shock proteins
HSR: heat-shock response
OPDA: 12-oxo-phytodienoic acid
opr3: *12-oxophytodienoic acid reductase 3*
PGA_1_: prostaglandin A_1_
PPA_1_: A_1_-phytoprostanes
RAD: radicicol
RES: reactive electrophile species
RNS: reactive nitrogen species
ROS: reactive oxygen species
SAT: short-term acquired thermotolerance
SAT1: serine acetyltransferase 1
