# Supplementary material for: Reactive electrophilic oxylipins trigger a heat stress-like response through HSFA1 transcription factors
Source: J Exp Bot. 2016 Oct 6;67(21):6139–48. doi: 10.1093/jxb/erw376 (PMC5100025; doi:10.1093/jxb/erw376)
Supplement: Supplementary Data [file supp_67_21_6139__index.html]

Reactive electrophilic oxylipins trigger a heat stress-like response through HSFA1 transcription factors — Reactive electrophilic oxylipins trigger a heat stress-like response through HSFA1 transcription factors — Supplementary Data 

# Reactive electrophilic oxylipins trigger a heat stress-like response through HSFA1 transcription factors

## Supplementary Data

Data files

- Supplementary\_table\_S1.pdf - Supplementary Data
