# Supplementary material for: Reactive electrophilic oxylipins trigger a heat stress-like response through HSFA1 transcription factors
Source: J Exp Bot. 2016 Oct 6;67(21):6139–48. doi: 10.1093/jxb/erw376 (PMC5100025; doi:10.1093/jxb/erw376)
Supplement: Supplementary Data [file supp_erw376_Supplementary_table_S1.pdf]

Journal of Experimental Botany

Reactive electrophilic oxylipins trigger a heat stress-like response through HSFA1 transcription factors

Miriam Muench, Chih-Hsuan Hsin, Elena Ferber, Susanne Berger, Martin J. Mueller

Supplementary Table S1

In silico analysis of OPDA and heat upregulated genes. Genes that have reported to be upregulated both by OPDA (75µM, 4h) and moderate heat (37°C, 1h) more than threefold are listed (Liu *et al.*, 2011; Mueller *et al.*, 2008). Under heat stress, upregulation of genes nos. 1 – 26 were found to be dependent on the transcription factors HSFA1 a, b, d and e (Liu *et al.*, 2011). The microarray data was extracted from the National Center for Biotechnology Information (NCBI) Gene Expression Omnibus under the accession nos. GSE10749 (OPDA-induced genes) and GSE26266 (heat-induced genes).

**No.**	**Locus**	**Names**	**Fold-induction by OPDA**	**Fold-induction by 37 C**
1	AT3G46230	AtHSP17.4	18,6	997
2	AT5G12030	AtHSP17.6A	30,5	988
3	AT1G53540	Unknown, HSP20-like chaperones superfamily protein	18,4	959
4	AT1G52560	Unknown, HSP20-like chaperones superfamily protein	52,3	655
5	AT4G27670	AtHSP21	8,3	516
6	AT5G12020	AtHSP17.6II	3,8	478
7	AT2G46240	AtBAG6 (Arabidopsis thaliana BCL-2-Associated Athanogene 6)	10	467
8	AT4G10250	AtHSP22.0	26,8	381
9	AT1G16030	AtHSP70B	28,4	369
10	AT2G29500	Unknown, HSP20-like chaperones superfamily protein	6,9	343
11	AT1G74310	AtHSP101	13	254
12	AT4G12400	HOP3	4	159

No.	Locus	Names	Fold-induction by OPDA	Fold-induction by 37 C
13	AT1G54050	Unknown, HSP20-like chaperones superfamily protein	4,8	142
14	AT2G32120	AtHSP70T-2	4,4	110
15	AT3G08970	TMS1, ATERDJ3A	3,2	107
16	AT2G47180	AtGOLS1	9,9	81,5
17	AT5G52640	AtHSP90-1, HSP81-1, HSP83	5,3	70,1
18	AT5G48570	ROF2, AtFKBP65	3,2	66,2
19	AT3G24500	MBF1C	3,6	51,8
20	AT5G05410	DREB2A	11,6	46,4
21	AT4G36990	AtHSF4, AtHSFB1, TBF1	4,3	28,1
22	AT4G25380	AtSAP10	8,9	33
23	AT2G24100	ASG1	3,1	16,7
24	AT3G28740	CYP81D11	71,8	3,6
25	AT2G29450	GSTU1 (Glutathione S-Transferase Tau 1), GSTU5, At103-1Am	3,1	13,1
26	AT5G59820	AtZAT12, RHL41 (Responsive to High Light 41)	6,5	10,5
27	AT5G04340	AtZAT6, C2H2 (Cold Induced Zinc Finger Protein 2), CZF2	4,1	13,4
28	AT1G64200	VHA-E3 (Vacuolar H^+-ATPase Subunit E Isoform 3)	3,9	3
29	AT2G23110	Unknown, Late Embryogenesis Abundant Protein, group 6	3,8	5,1
30	AT3G28210	SAP12 (Stress-Associated Protein 12), PMZ	10,4	34,4

References

Liu HC, Liao HT, Charng YY. 2011. The role of class A1 heat shock factors (HSFA1s) in response to heat and other stresses in Arabidopsis. *Plant Cell and Environment* 34, 738-751.

Mueller S, Hilbert B, Dueckershoff K, Roitsch T, Krischke M, Mueller MJ, Berger S. 2008. General detoxification and stress responses are mediated by oxidized lipids through TGA transcription factors in Arabidopsis. *Plant Cell* 20, 768-785.
